# Mechanism investigation of *Actinidia arguta* total flavone on gout via network pharmacology and *vivo* experiments pharmacological verification

**DOI:** 10.1515/biol-2025-1305

**Published:** 2026-06-18

**Authors:** Yilin Wang, Shuang Wang, Zhaoxia Li, Xi Chen, Chengyi Zhang, Xiaoxue Han, Limei Wan, Suiqiang Fan, Yiting Liu, Ziyan Guo, Xiaoqiang Huang, Xiaoze Xu, Xin Zhou, Bingfeng Xing

**Affiliations:** The First Affiliated Hospital of Guangdong Pharmaceutical University, Guangzhou, 510699, Guangdong, China; The Third Affiliated Hospital of Jinzhou Medical University, Jinzhou, 121000, Liaoning, China; Zhuhai Hospital of Integrated Traditional Chinese and Western Medicine, Zhuhai, 519000, Guangdong, China; College of Basic Medicine, Beihua University, Jilin, 132013, China; College of Pharmacy, Beihua University, Jilin, 132013, China; The First Affiliated Hospital, Sun Yat-sen University, Guangzhou, Guangdong, 510699, China

**Keywords:** *Actinidia arguta* total flavone, gout, hyperuricemia, network pharmacology, TNF

## Abstract

To clarify the therapeutic targets and signaling pathways of *Actinidia arguta* total flavone (AATF) in gout treatment, we integrated network pharmacology and *in vivo* experiments. Network pharmacology was applied to screen AATF’s anti-gout targets, construct protein-protein interaction (PPI) and drug-component-disease-target-pathway networks, and conduct gene ontology (GO) and Kyoto Encyclopedia of Genes and Genomes (KEGG) enrichment analyses. A dual gout rat model was established using potassium oxonate, adenine, and monosodium urate (MSU) crystals, followed by assessments including toe swelling measurement, hematoxylin-eosin (HE) staining of kidney and joint tissues, digital radiography (DR) imaging, detection of serum uric acid and xanthine oxidase (XOD) activity, enzyme-linked immunosorbent assay (ELISA) of inflammatory factors, and Western blot validation of key targets. Network pharmacology revealed that AATF modulates inflammatory responses and the tumor necrosis factor (TNF) pathway via core targets including albumin (ALB), TNF, interleukin-6 (IL-6), and tumor protein 53 (TP53). *In vivo* experiments showed that AATF significantly ameliorated renal and joint pathological damage, reduced serum uric acid/XOD activity, downregulated serum interleukin-1β (IL-1β), tumor necrosis factor-α (TNF-α), IL-6, cyclooxygenase-2 (COX-2) levels, and inhibited joint nuclear factor kappa B (NF-κB), extracellular signal-regulated kinase 1 (ERK1), matrix metalloproteinase 9 (MMP9) expression. Collectively, AATF exerts anti-gout effects through multi-target and multi-pathway mechanisms linked to the TNF signaling pathway, providing critical preliminary evidence for its preclinical development.

## 1 Introduction

Gout is an acute and chronic metabolic disease [1] caused by a mixture of genetic, environmental and lifestyle factors. It is caused by deregulated purine metabolism followed by elevated blood uric acid and urate deposition. Hyperuricemia, acute and chronic arthritis, severe joint pain and even joint deformity are the main clinical manifestations [2]. If not treated in time, kidney involvement can lead to uric acid nephrolithiasis, chronic interstitial nephritis, acute and chronic renal failure, uremia, and other serious consequences, joint involvement will lead to different degrees of joint degeneration and other chronic inflammation [3]. Hyperuricemia is not only one of the main risk factors for gout attacks, but also a risk factor for kidney disease and cardiovascular disease [4]. Nowadays, hyperuricemia is widely concerned as a public health issue. It is reported that the prevalence of hyperuricemia is 13.1–13.3 % in China, 11.3–47 % in the United States, 11.9–25.0 % in Europe, 26.8 % in Japan, and the prevalence is increasing year by year [5]. At present, the first-line treatment drugs for the treatment of gout are mainly allopurinol tablets, benzbromarone, colchicine, etc. Long-term use of these drugs to treat gout patients will lead to nausea, vomiting, diarrhea, renal function, and liver function damage [6].

Natural drugs are widely concerned because they have few side effects and can achieve multi-target therapy. In the kiwi family, *Actinidia arguta* is a wild large deciduous vine plant [7]. It naturally grows in Northeast China and other places and is used folkly for the treatment of a variety of diseases [8]. Its fruit is green, with a refreshing fragrance; it is soft and juicy, rich in nutrition, and can be used as both food and medicine. Hence, it has high economic and medical value [9]. At present, further investigations on the anti-inflammatory activity of *A. arguta* are almost blank around the world, which indicates its broad potential application prospect. Nonetheless, since *A. arguta* is a multi-target and multi-component agent, the underlying mechanisms are still unknown, despite the sufficient evidence supporting the beneficial effect of *A. arguta*.

Flavonoid is one of the main active ingredients of *A. arguta* [10]. This element is found to have physiological functions such as anti-oxidation, anti-inflammatory, antiviral, prevention and treatment to cardiovascular and cerebrovascular diseases, enhancing liver protection, and improving body immunity [11]. Our research group has studied the anti-inflammatory activity of hairy cherry total flavonoids in Synovial cells (RSC-364) and RAW264.7 cell of inflammatory model cells, which indicates that flavonoids have positive effects on anti-inflammatory activity, which deserves further study [12], 13]. Therefore, this study intends to explore and verify the mechanism of *A. arguta*’s gout treatment through network pharmacology and *in vivo* experiments, so as to provide and lay the experimental basis and theoretical basis for the development of new drugs for gout treatment in the future.

## 2 Materials and methods

### 2.1 Network pharmacological analysis of the molecular mechanism of *A. arguta* total flavone (AATF) treat gout

#### 2.1.1 Screening of core targets of AATF for the treatment of gout

The AATF were screened by Pubmed (https://pubmed.ncbi.nlm.nih.gov) and China National Knowledge Infrastructure (CNKI) (https://www.cnki.net) using the keywords “*A. arguta*” and “*A. arguta* total flavone” as keywords, screened the active ingredients of total flavonoids, combined with Traditional Chinese Medicine Systems Pharmacology Database and Analysis Platform (TCMSP) (https://old.tcmsp-e.com/tcmsp.php) and SwissTarget Prediction (https://swisstargetprediction.ch) to screen the AATF effective targets, and gene symbol conversion was performed by UniProt (https://www.uniprot.org). Apply GeneCards (https://www.genecards.org), online Mendelian Inheritance in Man (OMIM) (https://www.omim.org) and DrugBank online (https://go.drugbank.com) databases with “gout”, “gouty arthritis”, “gouty nephropathy”. The valid targets of AATF and gout were imported into VENNY 2.1 (https://bioinfogp.cnb.csic.es/tools/venny) to obtain the intersecting targets of AATF and gout, and the intersecting targets were imported into (search tool for the Retrieval of Interacting Genes/Proteins) STRING 2022 (https://string-db.org) database to obtain the protein interactions between the targets and construct PPI network, and applied the network topological properties plug-in in Cytoscape ver. 3.9.1 software to screen the core targets by topology complementation parameter analysis.

#### 2.1.2 GO analysis and KEGG analysis of AATF for gout

To obtain information on the biological processes, cellular components, molecular functions and pathways of AATF for the treatment of gout, we performed GO analysis and KEGG pathway analysis of the intersecting targets using the Database for Annotation, Visualization and Integrated Discovery (DAVID) (https://david.ncifcrf.gov) database. The drug-active component-target-disease-pathway network was constructed using Cytoscape ver. 3.9.1 and its topological properties, such as Degree Centrality (DC), Betweenness Centrality (BC) and Closeness Centrality (CC). Then to identify its key components and target targets, analyzations were done using the network analyzer plug-in. The topological properties of the network were evaluated by Network analyzer analysis to obtain key targets.

### 2.2 *In vivo* experimental validation

#### 2.2.1 Experiment animals

Sixty specific pathogen-free (SPF) grade Sprague Dawley (SD) rats, male, body mass (200 ± 20) g, were purchased from Changchun Yis Experimental Animal Technology Co Ltd [license number: SCXK (JL)2020-0003, SPF grade]. All animal care and procedures described in this study were approved by the ethics committee of Beihua University. Rats were housed in plastic rat cages with *ad libitum* diet and water, and the feed and bedding used were provided by Changchun Yis Experimental Animal Technology Co. The room temperature was (22 ± 2) °C, relative humidity was maintained within 55 ± 5 %, lights were switched on and off every 12 h, and relevant tests were conducted after one week of acclimatization.

**Ethical approval:** the research related to animal use has been complied with all the relevant national regulations and institutional policies for the care and use of animals, and has been approved by the Animal Ethics Committee of Beihua University (approval number: 20210507).

#### 2.2.2 Reagents and material

*A. arguta* total flavone (provided by the School of Pharmacy, Beihua University); Benzbromarone was purchased from Yichang Changjiang Pharmaceutical Co., LTD. (batch No.: 20211201); Potassium oxide salt (cargo number: P137112), adenine (cargo number: A108804), sodium urate salt (cargo number: U166391) and sodium carboxymethylcellulose sodium (cargo number: C83551) were purchased from Shanghai Aladdin Biochemical Technology Co., LTD.; Twain 80 was purchased from Tianjin Rugent Chemicals Co., Ltd.; Uric acid test kit (Lot #: 20211216), XOD activity kit (Lot #: 20211216), COX 2 test kit (batch number: 20211216) was purchased from Nanjing Jiancheng Biological Engineering Research Co., LTD.; Rat IL-1 β, TNF-α, and IL-6 test kits (20211021) were purchased from B & D Limited, USA. anti-β-actin antibody, anti-NFκB antibody, Anti-MMP9 antibody, anti-ERK1 antibody and horseradish peroxidase (HRP) goat anti-rabbit IgG were purchased from ABclonal.

#### 2.2.3 Preparation of mold-making agent

Adenine and potassium oxyzinate mixture: 5 g of adenine and 15 g of potassium oxyzinate were weighed in a 500 mL reagent bottle, mixed with an appropriate amount of 0.3 % sodium carboxymethyl cellulose (CMC-Na) solution, and then continued adding CMC-Na solution to fix the volume to 500 mL, sonicated, and shaken well to make the test solution into a uniform suspension, then stored in a refrigerator at 4 °C for backup.

Sodium urate suspension: MSU crystals were prepared by dissolving sodium urate salt (800 mg) in boiling water (155 ml) containing sodium hydroxide, adjusting the pH to 7.2 and gradually cooling the solution with stirring at room temperature. The crystals were collected by centrifugation (3,000 rpm, 4 °C, 5 min), evaporated and sterilized by heating in an infrared drying oven at 180 °C for 2 h. The MSU crystals were cooled and stored in sterile centrifuge tubes until use. Before use, 2 g of MSU crystals were weighed in Tween 80 to prepare a 20 g/L suspension of sodium urate [14].

#### 2.2.4 Animal modeling and handling

The experimental protocol was executed in a time-sequential manner.

– Days 0–6: all 60 SPF male SD rats were housed under standard conditions for adaptive feeding. On day 6, the rats were randomized into six groups (*n* = 10/group) based on the principle of consistent body weight distribution and similar health status: normal, control, benzbromarone (8.33 mg/kg), AATF100 (100 mg/kg), AATF200 (200 mg/kg), and AATF400 (400 mg/kg) [15].
– Days 7–21: hyperuricemia was induced in all groups except the normal group via daily intragastric administration of a mixed solution of adenine (100 mg/mL) and potassium oxonate (300 mg/mL) at a dose of 100 mg/100 g body weight; the normal group was gavaged with an equal dose of 0.3 % CMC-Na.
– Days 14–27: on the basis of modeling (Days 14–21) or routine feeding (Days 22–27), all groups were given corresponding therapeutic agents via intragastric gavage once daily at a fixed time. Specifically, the normal and control groups were gavaged with an equal volume of normal saline; the benzbromarone group received benzbromarone solution at a dose of 8.33 mg/kg; the AATF groups were treated with AATF solutions at doses of 100, 200, and 400 mg/kg, respectively.
– Day 20: blood samples were collected from the retro-orbital venous plexus at 8:00 a.m. and 14:00 to assess the dynamic changes in serum uric acid levels during treatment.
– Day 25: gouty arthritis was induced in all groups except the normal group by intra-articular injection of 0.2 mL of 20 g/L monosodium urate suspension into the right hind ankle using a no. Six injection needle; the normal group was injected with an equal volume of normal saline [16].
– Day 27: after the last intragastric administration, all rats were euthanized, and relevant tissue and fluid samples were collected for subsequent detection.

#### 2.2.5 Detection of the degree of foot swelling and gait observation

To reduce the interference of the experimental results, the swelling was measured twice for each joint and the swelling was calculated. The calculation formula was as follows:

$$S\text{welling }\left(\text{mL}\right)=v\text{olume after molding }\left(\text{mL}\right)-v\text{olume before molding }\left(\text{mL}\right)$$

After modelling, the gait grade was assessed at 24 h and 48 h. Gait observation was assessed according to the following criteria [17]: (gait grading) grade 1: normal walking; grade 2: slight limp with slight flexion of the subject’s lower limb; grade 3: limp with the subject’s lower limb just touching the ground; grade 4: severe limp with the subject’s lower limb leaving the ground and walking on three feet.

#### 2.2.6 Biochemical indexes

Blood samples were collected from each group on day 20 of the experiment at the inner canthus plexus to detect uric acid levels. On the 27th day of the experiment, serum was collected from the abdominal aorta to detect serum uric acid and XOD enzyme activity. On the 27th day of the experiment, urine was collected to detect urine uric acid content. The above indicators were measured according to the kit method.

#### 2.2.7 Hematoxylin-eosin (HE) staining of kidney and joint tissues

The kidneys were fixed in 10 % formalin. The organs were cut into applicable tissue blocks, washed, dehydrated and embedded to make 5 μm tissue paraffin sections and stained with HE. The joints were fixed in 10 % formalin, decalcified with 10 % concentrated nitric acid, trimmed into applicable tissue blocks, cleaned, dehydrated and embedded, made into 5 μm tissue paraffin sections and then stained with HE. The stained specimens were photographed and analyzed with a bioimaging microscope.

#### 2.2.8 Imaging examination

On the 26th day of the experiment, i.e., the 24th after the injection of MSU crystal suspension into the ankle joint of rats, rats were given an intraperitoneal injection of 3 % pentobarbital sodium 1 ml/kg to anesthetize the rats, and the rats were placed under a Digital Radiography (DR) imager for joint photographic analysis.

#### 2.2.9 ELISA for detection of inflammatory factors in serum

On the 27th day of the experiment, blood samples were collected from each group via abdominal aortic blood collection. Subsequently, the samples were left at room temperature for 30 min, then centrifuged at 3,000 r/min for 10 min at 4 °C, and the upper – layer serum was extracted. The serum levels of TNF-α, IL-6, IL-1β, and COX-2 were measured by enzyme immunoassay following the instructions of the kit.

#### 2.2.10 Apply western bloting to verify the targets

Total proteins were extracted from joint tissues using radioimmunoprecipitation assay (RIPA) lysis buffer supplemented with protease and phosphatase inhibitors, and protein concentrations were quantified via the bicinchoninic acid (BCA) protein assay kit. For electrophoresis, 12 % sodium dodecyl sulfate-polyacrylamide gel electrophoresis (SDS-PAGE) gels comprising stacking and separating gels were first prepared, after which equal amounts of protein (50 μg per lane) were loaded and separated at a constant voltage of 70 V for 30 min followed by 90 V for 1.5 h. Separated proteins were subsequently transferred onto 0.45 μm polyvinylidene fluoride (PVDF) membranes at 100 V for 1 h, and the membranes were blocked with 5 % non-fat milk for 1 h at room temperature to eliminate non-specific binding. The blocked membranes were incubated overnight at 4 °C with primary antibodies diluted in Tris-buffered saline with Tween 20 (TBST), including rabbit anti-mouse β-actin (1:1,000), rabbit anti-mouse NF-κB (1:1,000), rabbit anti-mouse ERK1 (1:1,000), and rabbit anti-mouse MMP9 (1:1,000). After primary antibody incubation, the membranes were rinsed with TBST three times for 15 min, 10 min, and 5 min, respectively, then incubated with HRP-conjugated goat anti-rabbit IgG secondary antibody (1:5,000) for 2 h at room temperature, followed by re-washing with TBST using the same time intervals as described above. Protein bands were visualized using an enhanced chemiluminescence (ECL) enhanced chemiluminescence kit, with β-actin serving as the internal reference, and the relative gray values of the bands were quantified using Image J software. All experiments were independently repeated at least three times to ensure the reproducibility of results.

### 2.3 Statistical analysis

Data were expressed as mean ± standard deviation (mean ± SEM), and one-way analysis of variance was performed using GraphPad Prism 9. The difference among the means was considered to be significant if *P* < 0.05.

## 3 Results

### 3.1 Network pharmacology to study the molecular mechanism of AATF in the treatment of gout

#### 3.1.1 PPI network construction and the screening of core targets

**Figure 1:**
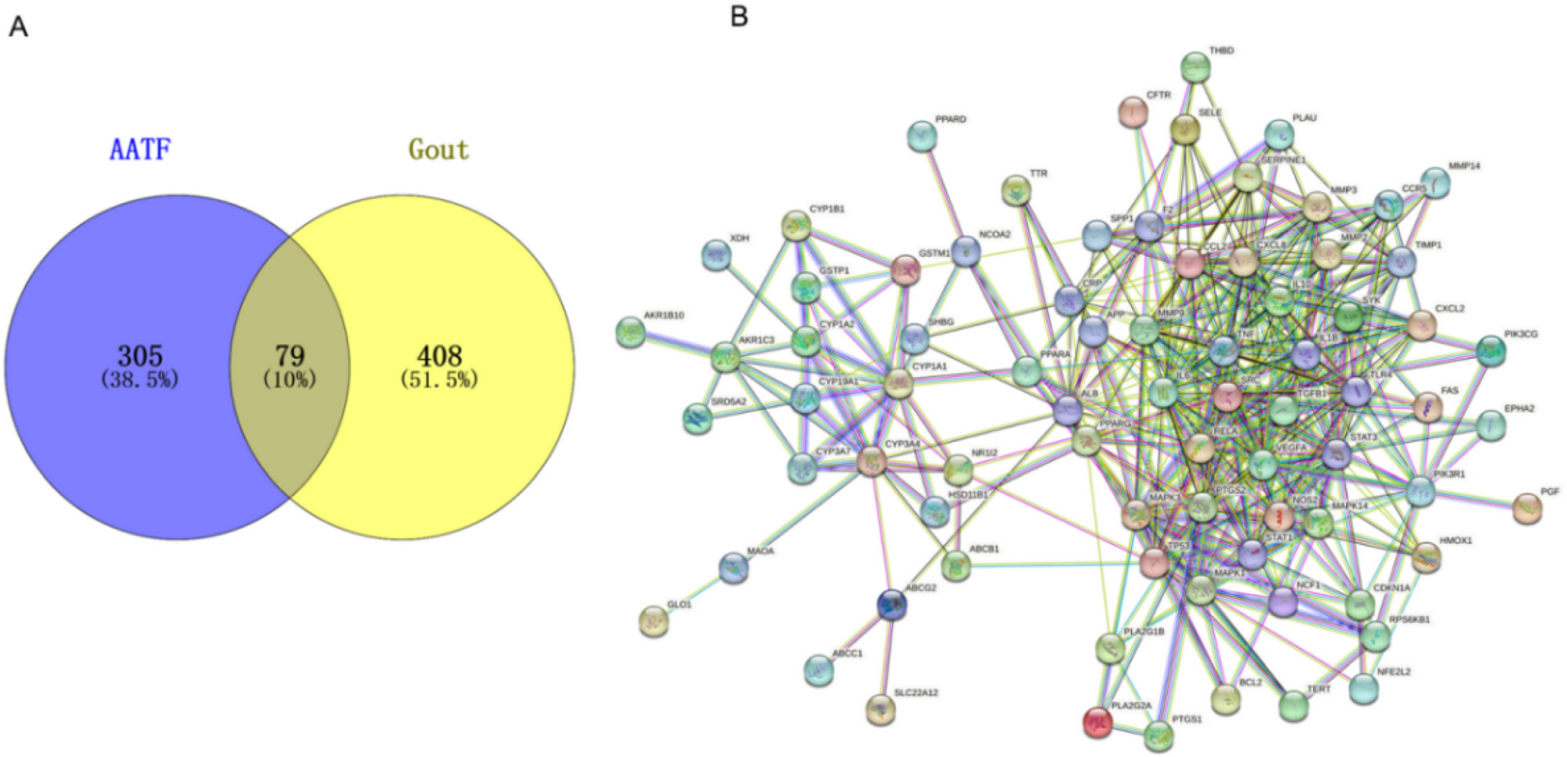
AATF treatment for gout intersection and its protein interaction relationship. (A) The venn diagram of targets between AATF-related targets and gout-related; (B) PPI network of AATF for treating gout targets.

By searching Pubmed and CNKI database, Absorption, Distribution, Metabolism, Excretion (ADME) pharmacokinetics (OB > 30 %, DL > 0.18), which were *quercetin* [18], 19], *luteolin* [19], *(−)*-*catechin* [20], *kaempferol* [21], *(−)*-*epicatechin* [19], 22], *(−)*-*epigallocatechin-3-gallate* [19], *(+)*-*catechin* [23] (Table 1). And combining TCMSP and SwissTarget Prediction database to select 384 targets associated with AATF active components, applying the Gene Card, OMIM and the DrugBank database screened out 487 targets associated with the disease, import of effective targets of drugs and disease into VENNY2.1 yields the intersection of targets of AATF and gout 79, import of the two intersection targets into the STRING 2022 database, set the species as a human, higher confidence of 0.7, FDR < 0.05, intertarget protein interaction relationships were obtained as shown here, a total of 79 nodes were obtained, side of 409 bars, average node degree of 10.4, the average local clustering coefficient was 0.593, PPI enrichment *P*-value:<1.0e−16 (Figure 1A and B).

**Table 1:** The reactive ingredients of AATF.

Ingredient	OB/%	DL	Number
*quercetin*	46.43	0.28	AATF1
*luteolin*	36.16	0.25	AATF2
*(*−*)-catechin*	49.68	0.24	AATF3
*kaempferol*	41.88	0.24	AATF4
*(*−*)-epicatechin*	28.93	0.24	AATF5
*(*−*)-epigallocatechin-3-gallate*	55.09	0.77	AATF6
*(*+*)-catechin*	54.83	0.24	AATF7

**Figure 2:**
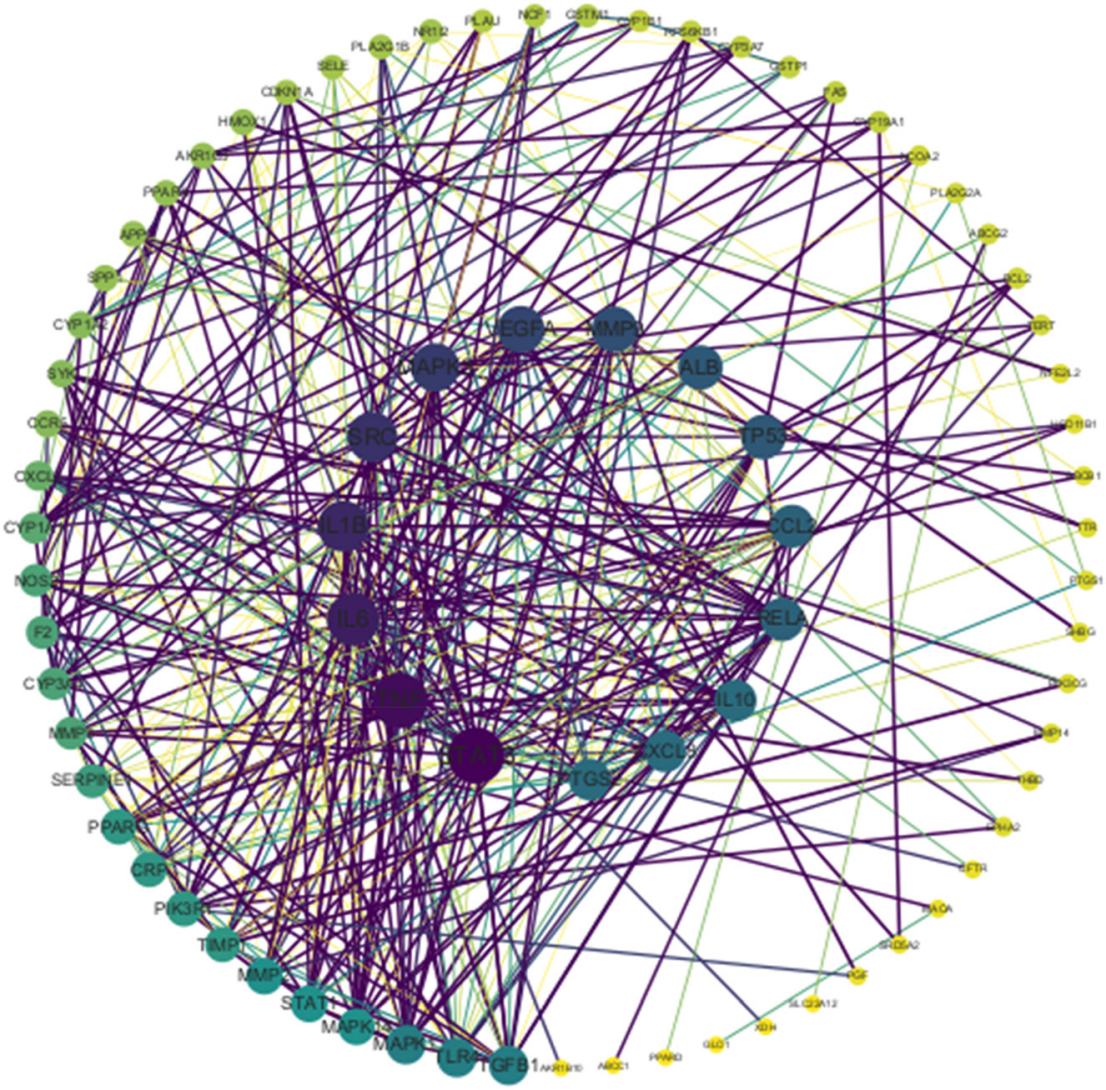
Analysis of network extension properties of AATF for gout targets. AATF: *Actinidia arguta* total flavone.

Apply the network topological properties plug-in in Cytoscape ver. 3.9.1 software for the 79 targets (DC values, BC values, CC values), as shown in the figure, the degree value was positively correlated with the degree of the protein core, the darker the color is, the larger the graph indicates that the higher the degree of the protein core; the combined-score indicates the support of the data, the thicker the connection is, a darker color indicates a stronger interaction between the two proteins (Figure 2). According to the top 15 targets of the network topology properties (DC values, BC values, CC values), the 15 targets in the middle of the PPI network served as the core targets for the treatment of total flavonoids in gout (Table 2).

**Table 2:** Core target of AATF in the treatment of gout.

Gene	Protein	Degree	Betweenness	Closeness
STAT3	Signal transducer and activator of transcription 3	31	263.31815	0.5310344
TNF	Tumor necrosis factor	30	275.77838	0.5310344
IL6	Interleukin 6	28	152.64156	0.5167785
IL1B	Interleukin 1 beta	27	144.93027	0.5099337
SRC	SRC proto-oncogene, non-receptor tyrosine kinase	26	658.77313	0.5133333
MAPK3	Mitogen-activated protein kinase 3	25	342.90738	0.5167785
VEGFA	Vascular endothelial growth factor A	24	282.1359	0.5065789
MMP9	Matrix metallopeptidase 9	23	137.62657	0.503268
ALB	Albumin	22	1,163.8678	0.55
TP53	Tumor protein p53	22	490.74405	0.5167785
RELA	RELA proto-oncogene, NF-κB subunit	21	88.29851	0.4782608
CCL2	C–C motif chemokine ligand 2	21	68.840195	0.4782608
IL10	Interleukin 10	20	46.765934	0.4666666
CXCL8	C-X-C motif chemokine ligand 8	20	53.295258	0.4666666
PTGS2	Prostaglandin-endoperoxide synthase 2	20	241.10048	0.4842767

#### 3.1.2 GO and KEGG enrichment analysis

**Figure 3:**
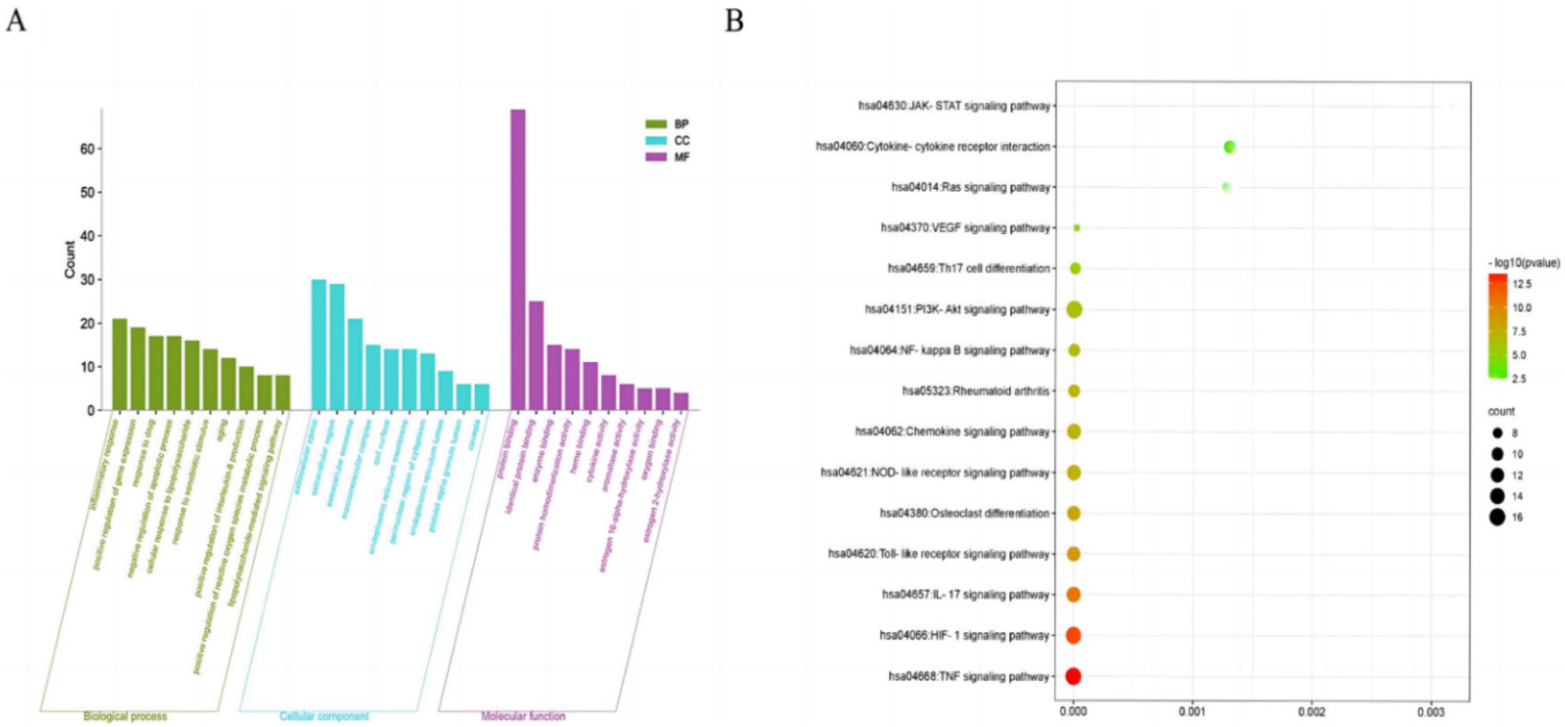
GO and KEGG enrichment analysis of AATF for gout. (A) The top 10 significantly enriched terms in biological process (BP), cellular component (CC) and molecular function (MF); (B) the top 15 significantly enriched terms in KEGG pathway (KEGG).

The GO and KEGG analysis of the intersection targets using the DAVID database, found that AATF in the treatment of gout after inflammatory response, positive regulation of gene expression and response to drug et al. 503 biological processes; extracellular space, and extracellular region and extracellular exosome et al. 44 cellular components; protein binding, and identical protein binding and enzyme binding et al. 100 molecular functions, and the top 10 were visualized according to the *P* value (Figure 3A). At the same time, we found that the targets mapped to 150 signaling pathways, excluding unrelated pathways such as “cancer pathway,” “hepatitis B” “and” “prostate cancer,” and obtained the top 15 pathways with the largest number of enriched targets. The results showed that most of these signaling pathways are involved in inflammatory responses, such as TNF, Phosphatidylinositol 3-Kinase-Protein Kinase B (PI3K-Akt), Mitogen-Activated Protein Kinase (MAPK), NF-kappa B, Nucleotide-binding Oligomerization Domain-like (NOD-like) receptor, and toll-like receptor signaling (Figure 3B).

#### 3.1.3 “Drug-active component-disease-target-pathway” network construction

**Figure 4:**
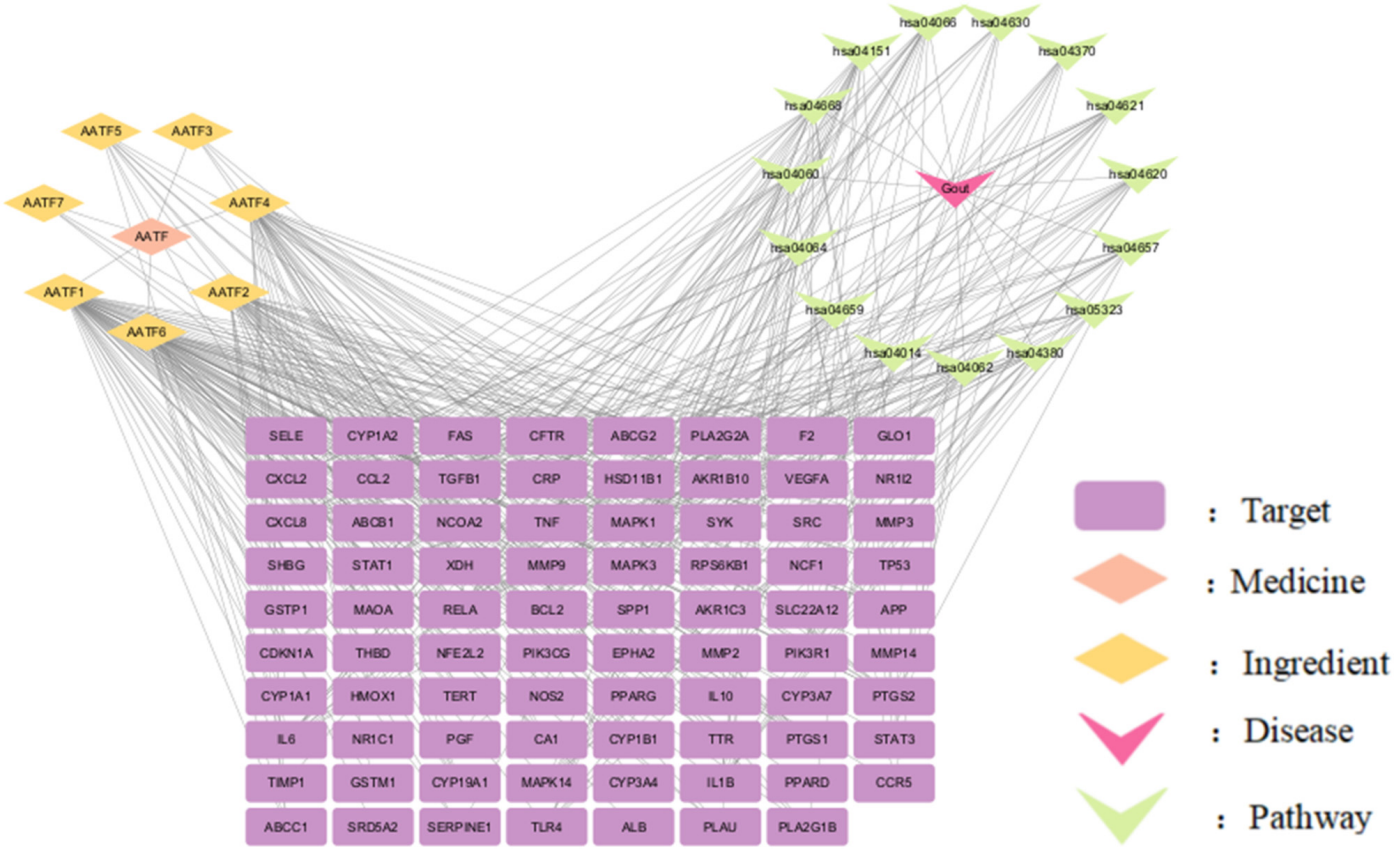
“Drug-active component-disease-target-pathway” network.

The drug component-disease-target network for gout treatment was constructed using Cytoscape ver. 3.9.1. The network consists of 103 nodes (7 active components, 79 targets, and 15 pathways) and 394 edges. The orange diamond represents the active component, the purple square indicates the potential target, and the green V quadrangle indicates the pathway. AATF 1–7 represents *quercetin*, *luteolin*, *(−) -catechin, kaempferol*, *(−) -epicatechin*, *(−) -epigallocatechin-3-gallate*, *(*+*) -catechin*, respectively. Topological parameters of the “drug-active component-disease-target-pathway” network, including DC, BC, and CC, were analyzed using the network analyzer plugin of Cytoscape3.9.1. We showed that the average DC of this active component was 29, with an average BC of 1,243.819, and an average CC of 0.459309. DC, BC and CC values were higher than average: *quercetin* (DC = 61, BC = 3, 688.306, CC = 0.625767); *(*−*)-epigallocatechin-3-gallate epigallocatechin-3-gallate* (DC = 48, BC = 2,909.4, CC = 0.539683), which may be the key active components for AATF treatment of AATF. However, the mean values of DC, BC, and CC of the target sites were 5, 51.48423647, and 0.425452132, respectively, and 23 targets showed high topological values: nuclear factor-kappa B p65 Subunit (RELA), IL6, Mitogen-Activated Protein Kinase 1 (MAPK1), Phosphoinositide-3-Kinase Regulatory Subunit 1 (PIK3R1), TNF, Mitogen-Activated Protein Kinase 3 (MAPK3), Prostaglandin-Endoperoxide Synthase 2 (PTGS2), IL1B, C-X-C Chemokine Ligand 2 (CXCL2), C–C Chemokine Ligand 2 (CCL2), Vascular Endothelial Growth Factor A (VEGFA), B-Cell Lymphoma 2 (BCL2), C-X-C Chemokine Ligand 8 (CXCL8), Signal Transducer and Activator of Transcription 1(STAT1), pleen Tyrosine Kinase (SYK), Prostaglandin-Endoperoxide Synthase 1 (PTGS1), MMP9, Cyclin-Dependent Kinase Inhibitor 1A (CDKN1A), MMP3, Peroxisome Proliferator-Activated Receptor Gamma (PPARG), Proto-Oncogene Tyrosine-Protein Kinase SRC (SRC), Phosphoinositide-3-Kinase Catalytic Subunit Gamma (PIK3CG), IL10; the mean values of the pathway for DC, BC, and CC were 13, 111.3512457, and 0.387453082, respectively, five pathways showed high colonization values: hsa04668 (TNF signaling pathway), hsa04151(PI3K-Akt signaling pathway), hsa04066(HIF-1 signaling pathway), hsa04062 (Chemokine signaling pathway chemokine signaling pathway), hsa04380 (Osteoclast differentiation osteoclast differentiation) (Figure 4).

### 3.2 *In vivo* experiments to verify the molecular mechanism of AATF treatment of gout in rats

#### 3.2.1 Effect of AATF on the degree and gait of joint foot swelling in the rat double model of gout

**Figure 5:**
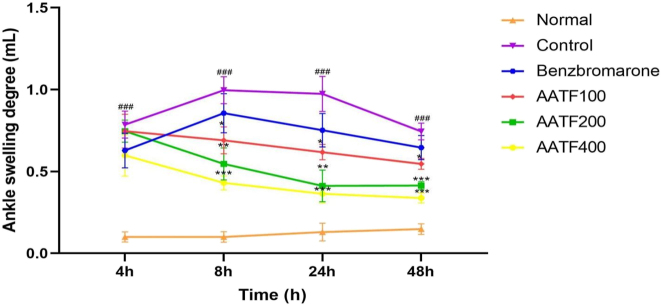
Effect of AATF on toe swelling in a double model of gout. The number of samples in each group was 10. All data represent the mean ± SD compared with the normal group, ###: *P* < 0.001, compared with the control group, *: *P* < 0.05, **: *P* < 0.01, ***: *P* < 0.001.

The gait classification of gout rats was observed at 24 h and 48 h after the mold formation of gouty arthritis. Compared to the normal group, gait grade showed significant differences in the control group (*P* < 0.001). Compared with the control group, gait grade there was no significant difference, and the movement gait of gout rats in the AATF100, AATF200 and AATF400 group improved significantly, and increased with the increase of the total flavonoid dose of soft jujube macaques (Table 3). On the 4 h, 8 h, 24 h and 48 h after mold making, compared with the normal group, the control swelling increased significantly in each time period, not the foot swelling at each time node in benzbromarone group, AATF100, AATF200 and AATF400 group at 8 h, 24 h and 48 h (*P* < 0.05, *P* < 0.01 or *P* < 0.001), and there was no significant difference, but suppressed the foot swelling trend when compared with the control group (Figure 5).

**Table 3:** Effect of AATF on gait in the double model of gout rats.

Groups	Dose (mg/kg)	24 h	48 h
Normal	–	1.10 ± 0.32^a^	1.00 ± 0.00^a^
Control	–	3.50 ± 0.53^a^	3.00 ± 0.67^a^
Benzbromarone	8.33	3.00 ± 0.82^a^	2.70 ± 0.48
AATF100	100	2.90 ± 0.57^b^	2.20 ± 0.79^b^
AATF200	200	2.40 ± 0.84^c^	1.80 ± 0.67^c^
AATF400	400	1.80 ± 0.79^d^	1.70 ± 0.79^d^

The number of samples in each group was 10. All data represent the mean ± SD Compared with the normal group, ^a^ *P* < 0.001, Compared with the control group, ^b^ *P* < 0.05, ^c^ *P* < 0.01, ^d^ *P* < 0.001.

#### 3.2.2 AATF effect on joint imaging in double model of gout

**Figure 6:**
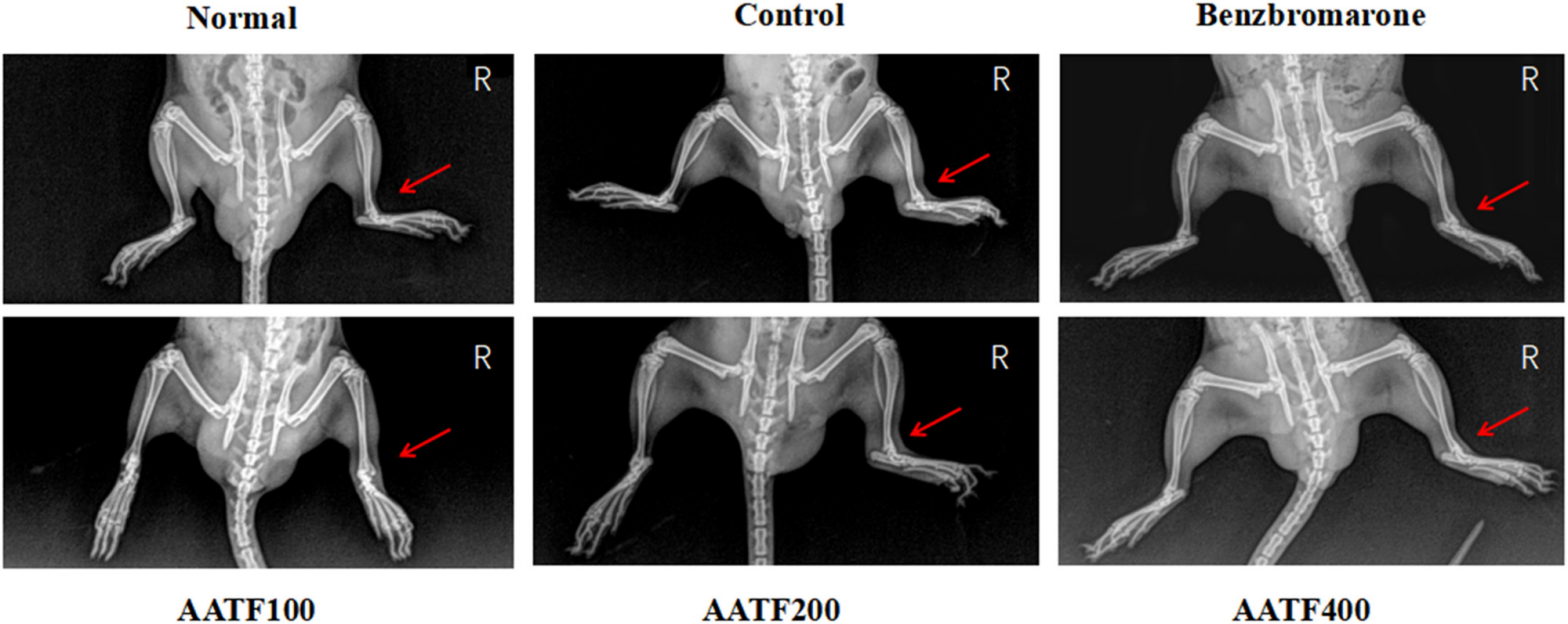
DR imaging of the double model of gout rats. The red arrow indicates the mold foot.

The normal group did not make a model for gout, and no swelling in the right and right hind feet in the control group; compared with the one in the AATF each dose groups, the swelling was observed, and the swelling was less than that of the control group (Figure 6).

#### 3.2.3 AATF effect on serum uric acid content, urine uric acid content and XOD enzyme activity in the double model of gout rats

**Figure 7:**
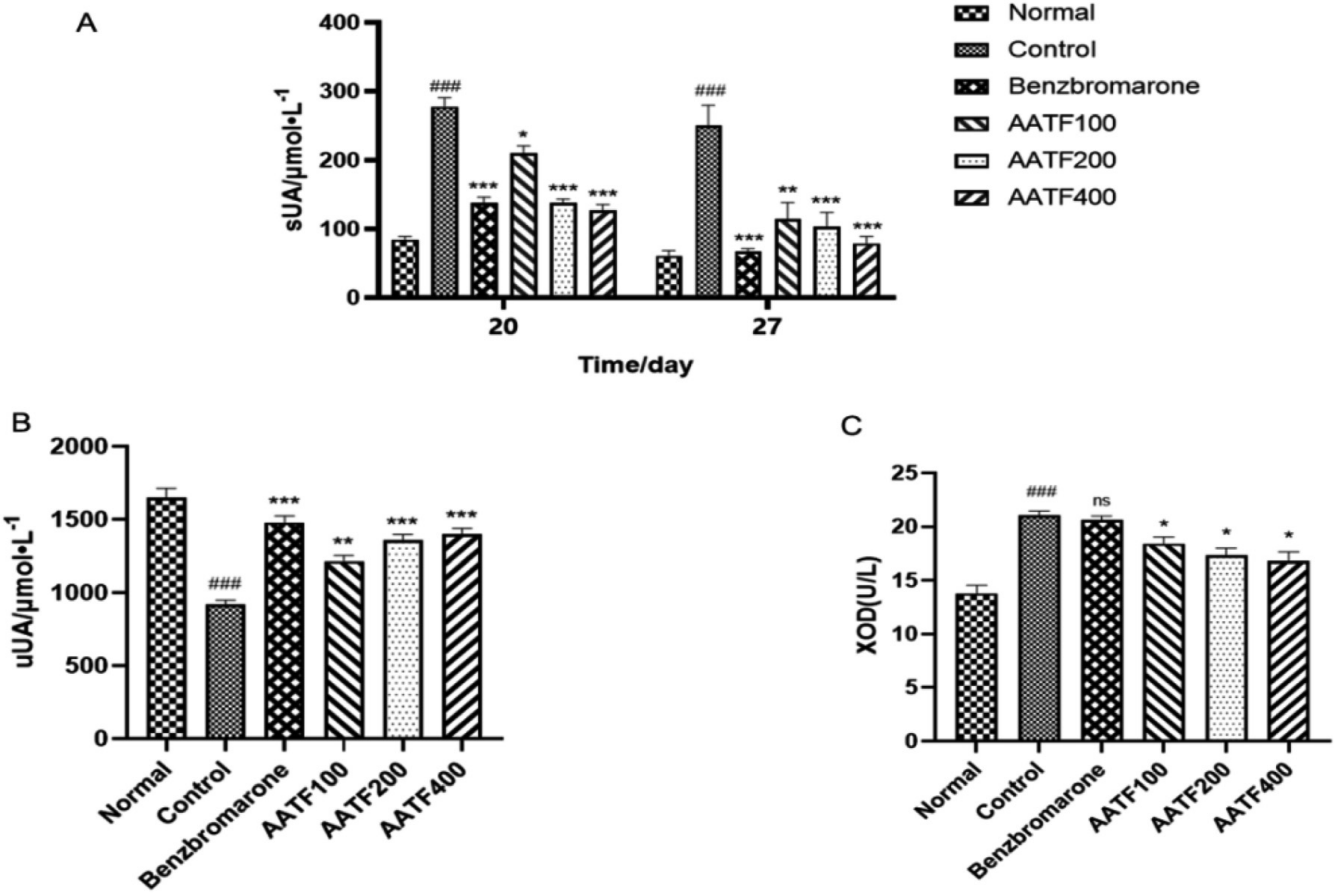
Effects of AATF on uric acid metabolism in double model of gout rats. (A) Effects of AATF on serum UA in double model of gout rats; (B) effects of AATF on urine UA in double model of gout rats; (C) effects of AATF on XOD in double model of gout rats. The number of samples in each group was 10. All data represent the mean ± SD compared with the normal group, ###: *P* < 0.001, compared with the control group, *: *P* < 0.05, **: *P* < 0.01, ***: *P* < 0.001, ns: not significant.

Successful model establishment was confirmed by the marked elevation of serum uric acid (sUA) levels in the control group on both day 20 and day 27 (*P* < 0.001 vs. normal group), and compared with the control group, all treatment groups exhibited significant reductions in sUA levels, with benzbromarone decreasing sUA by 24.45 % on day 20 and 29.06 % on day 27, AATF100 decreasing sUA by 40.44 % on day 20 and 45.01 % on day 27, AATF200 decreasing sUA by 45.35 % on day 20 and 50.10 % on day 27, and AATF400 decreasing sUA by 50.26 % on day 20 and 55.22 % on day 27 (*P* < 0.01 for all comparisons); these results demonstrate that both benzbromarone and AATF exerted a dose-dependent uric acid-lowering effect, with AATF showing greater efficacy at higher doses (Figure 7A; Table 4).

**Table 4:** Effects of AATF on sUA levels in double model of gout rats on day 20 and day 27.

Groups	Dose (mg/kg)	sUA (µmol/L, day 20)	sUA (µmol/L, day 27)
Normal	–	84.70 ± 14.39^a^	64.07 ± 24.44^a^
Control	–	277.97 ± 49.11^a^	256.86 ± 94.74^a^
Benzbromarone	8.33	138.30 ± 29.06^d^	97.30 ± 11.74^d^
AATF100	100	151.17 ± 39.23^b^	115.25 ± 23.44^c^
AATF200	200	127.91 ± 32.02^d^	109.74 ± 12.02^d^
AATF400	400	108.74 ± 14.94^d^	102.13 ± 12.16^d^

The number of samples in each group was 10. All data represent the mean ± SD Compared with the normal group, ^a^ *P* < 0.001, Compared with the control group, ^b^ *P* < 0.05, ^c^ *P* < 0.01, ^d^ *P* < 0.001.

The bar graph shows that the control group exhibited a 44.1 % decrease in urinary uric acid (uUA) concentration relative to the normal group (*P* < 0.001), confirming successful model establishment. All treatment groups, including benzbromarone and AATF at all doses, demonstrated significant increases in uUA concentration compared with the control group: benzbromarone (60.4 % increase, *P* < 0.001), AATF100 (31.8 % increase, *P* < 0.05), AATF200 (47.1 % increase, *P* < 0.001), and AATF400 (52.0 % increase, *P* < 0.001). These findings indicate that under the current experimental conditions, both agents elevated uUA concentration in a dose-dependent manner, suggesting enhanced uric acid excretion (Figure 7B; Table 5).

**Table 5:** Effects of AATF on uUA levels and XOD activity in double model of gout rats.

Groups	Dose (mg/kg)	uUA (mol/L)	XOD activity (U/L)
Normal	–	1,649.6 ± 202.0^a^	13.81 ± 2.33^a^
Control	–	922.2 ± 83.8^a^	21.03 ± 1.99^a^
Benzbromarone	8.33	1,479.6 ± 141.4^d^	20.95 ± 1.69^ns^
AATF100	100	1,215 ± 119.00^c^	18.41 ± 1.93^b^
AATF200	200	1,356.6 ± 127.4^d^	17.02 ± 2.17^b^
AATF400	400	1,402.2 ± 116.8^d^	15.79 ± 2.69^b^

The number of samples in each group was 10. All data represent the mean ± SD compared with the normal group, ^a^ *P* < 0.001, Compared with the control group, ^b^ *P* < 0.05, ^c^ *P* < 0.01, ^d^ *P* < 0.001, ns: not significant.

On day 27 of the experiment, the XOD enzyme activity in the serum of each group was tested, and compared with the normal group, the serum XOD enzyme activity of the control group was significantly increased (*P* < 0.05). Compared with the control group, the XOD enzyme activity in the benzbromarone group was no statistical difference. Compared with the control group, the XOD enzyme activity in the AATF each dose groups were significantly decreased (*P* < 0.05), and it was dose-dependent with the increase of the dose (Figure 7C; Table 5).

#### 3.2.4 AATF effect on kidney and joint histopathology in the rat double model of gout

**Figure 8:**
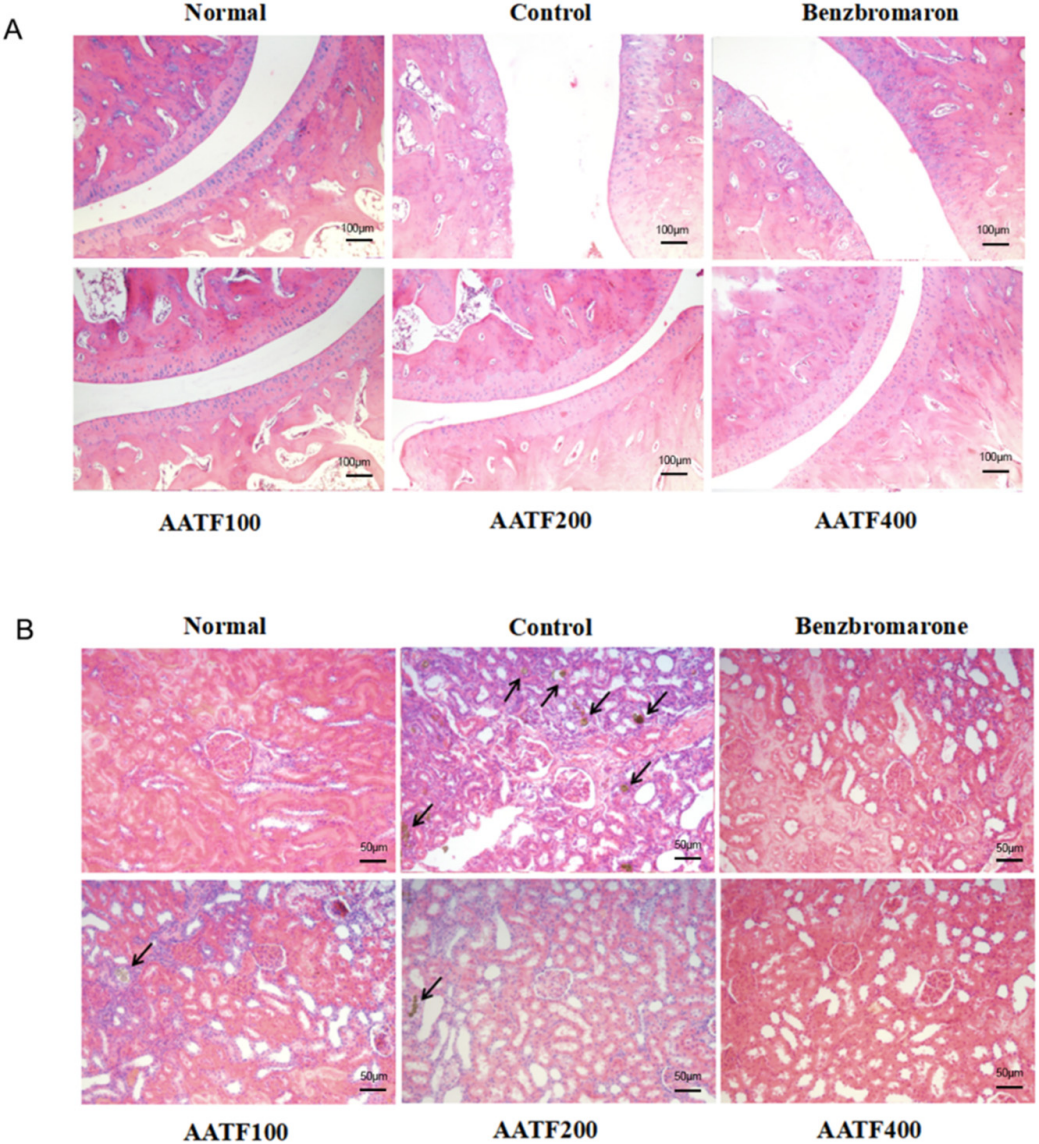
Effect of AATF on histopathology of gout double model rats. (A) Effect of AATF on joint histopathology of gout double model rats. Images were magnified by 100×; (B) effect of AATF on kidney histopathology of gout double model rats. Images were magnified by 200×. Black arrows indicate the urate crystals.

Clear ankle joint synovial tissue structure in the normal group, no or little infiltration of inflammatory cells, no significant subsynovial cartilage tissue destruction was observed; significant inflammatory response in the synovial tissue in the model group, disorder of the synovial tissue, the cell proliferation was evident, cellular congestion, with visible oedema and necrosis of partial synovial tissue, accompanied by a large number of inflammatory cell infiltration; joint synovial tissue hyperplasia was more in benzbromarone, AATF100 groups and ATTF200, synovial cells and surrounding tissues have different degrees of congestion, edema and inflammatory cell infiltration; the synovial tissue was essentially normal or mild hyperplasia in the AATF400 group, with mild congestion and oedema, a small infiltration of inflammatory cells was seen (Figure 8A).

In the normal group, glomeruli and tubules were neat and plump, with no neutrophil infiltration or brown crystal deposits in the tissue; numerous scattered brown crystal crystals in the control group, massive neutrophil infiltration in the renal tissue, glomerular vacuolar degeneration in the AATF100 and AATF200 dose groups, and a small amount of brown crystal deposition. A small extent of neutrophil infiltration and glomerular atrophy or vacuolar degeneration are observed, and brown crystal deposition were seen in the benzbromarone and AATF400 groups (Figure 8B).

#### 3.2.5 AATF effect on the content of TNF-α, IL-1β, IL-6, and COX-2 in the serum of a rat double model of gout

Compared with the normal group, the serum TNF-α, IL-1 β, IL-6 and COX-2 content witnessed a significant increase in the control group (*P* < 0.05). Compared with the control group, the levels of inflammatory factors in each treatment group decreased to varying degrees. Specifically, the positive control benzbromarone group (8.33 mg/kg) exhibited robust anti-inflammatory activity, with IL-1β, TNF-α, IL-6, and COX-2 levels reduced by 43.5 %, 59.8 %, 45.1 %, and 24.9 %, respectively. the AATF100 group only showed mild anti-inflammatory effects, with the above indicators decreased by 10.5 %, 8.8 %, 19.2 %, and 17.1 %, respectively. When the dose was increased to 200 mg/kg, the anti-inflammatory activity of AATF was significantly enhanced, with the reduction rates of each index increased to 25.2 %, 29.9 %, 32.0 %, and 23.1 %. At the AATF400 group, the inhibitory effect of AATF on inflammatory factors was comparable to that of the positive control drug, with IL-1β, TNF-α, IL-6, and COX-2 levels decreased by 37.6 %, 57.9 %, 44.8 %, and 26.1 %, respectively, showing a clear dose-dependent pattern (Table 6).

**Table 6:** Effects of AATF on IL-1β, TNF-α, IL-6 and COX-2 content in double model of gout rats.

Groups	Dose (mg/kg)	IL-1β (pg/ml)	TNF-α (pg/ml)	IL-6 (pg/ml)	COX-2 (ng/ml)
Normal	–	57.99 ± 11.82^ab^	46.62 ± 6.00^ab^	171.11 ± 40.07^ab^	2.80 ± 0.28^ab^
Control	–	139.16 ± 7.25^a^	368.73 ± 23.11^a^	383.23 ± 110.01^b^	4.90 ± 0.50^b^
Benzbromarone	8.33	78.66 ± 5.91^ac^	148.37 ± 24.19^c^	210.45 ± 90.18^c^	3.68 ± 0.24^c^
AATF100	100	124.51 ± 6.81^c^	336.20 ± 13.25^c^	309.78 ± 99.45^c^	4.06 ± 0.25^c^
AATF200	200	104.08 ± 5.55^c^	258.40 ± 15.08^c^	260.43 ± 70.34^c^	3.77 ± 0.26^c^
AATF400	400	86.87 ± 7.37^c^	155.26 ± 25.10^c^	211.64 ± 53.23^c^	3.62 ± 0.15^c^

All data represent the mean ± SD compared with the normal group, ^a^ *P* < 0.05, ^b^ *P* < 0.01, Compared to the control group, ^c^ *P* < 0.05.

#### 3.2.6 Effect of soft jujube kiwi total flavonoids on the amount of NF-κB, MMP 9 and ERK 1 expression in a double model of gout rats

**Figure 9:**
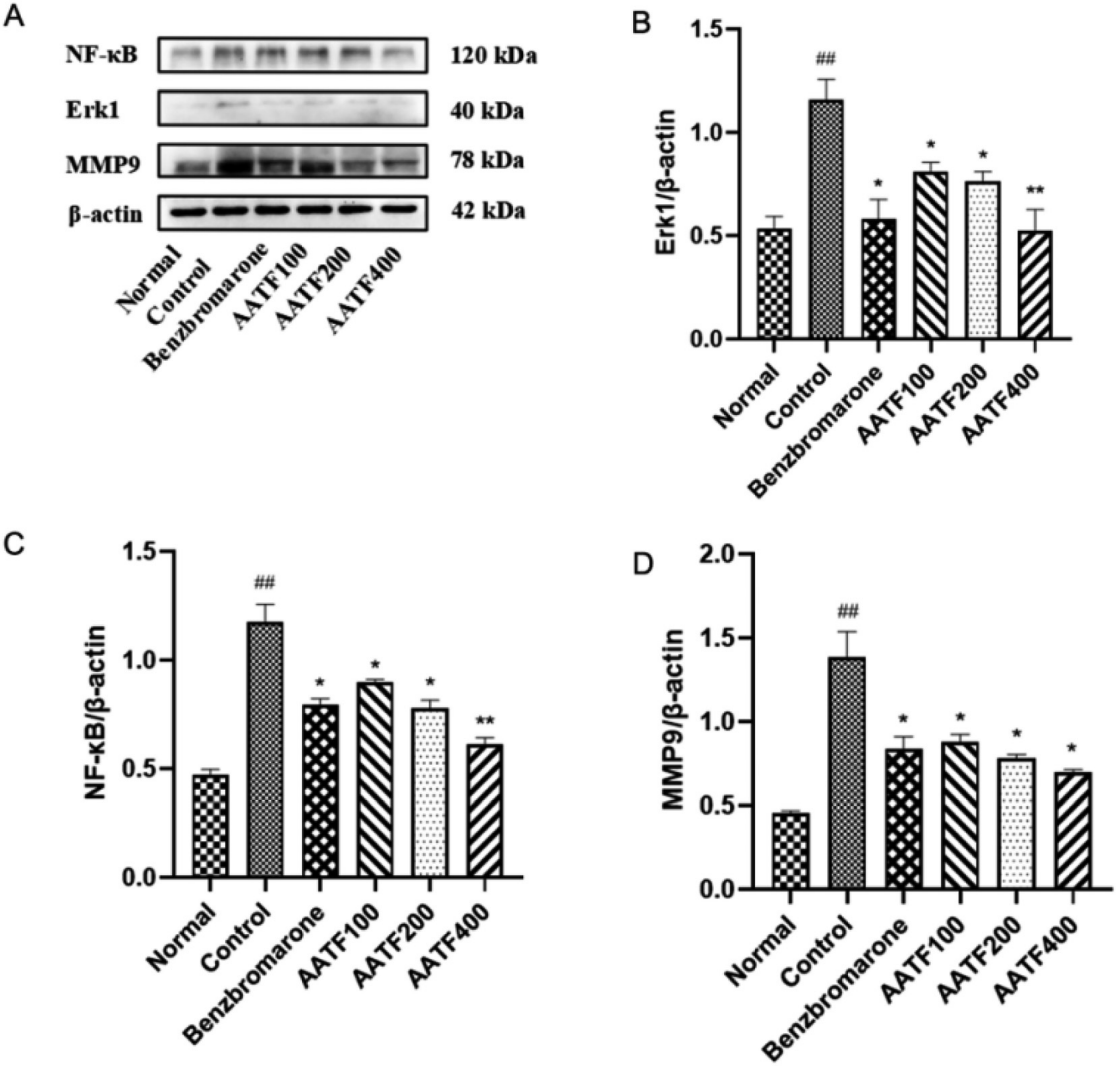
Effects of AATF on the expression of gout genes. (A) Representative western blot bands. (B) Effects of AATF on Erk1 relative protein expression levels in synovium of double model of gout rats. (C) Effects of AATF on NF-κB relative protein expression levels in synovium of double model of gout rats. (D) effects of AATF on MMP9 relative protein expression levels in synovium of double model of gout rats. The number of samples in each group was 10. All data represent the mean ± SD compared with the normal group, ##: *P* < 0.01, compared to the control group, *: *P* < 0.05, **: *P* < 0.01.

Compared with the normal group, NF-κB, MMP 9 and ERK 1 protein expression levels significantly increased in control group rats (*P* < 0.05). Compared with the control group, the AATF100, AATF200 and AATF400 group NF-κ B, MMP 9 and ERK 1 protein expression levels significantly decreased (*P* < 0.05 or *P* < 0.01), it presents the dose-dependent (Figure 9A–D).

## 4 Discussion

Hyperuricemia is one of the main factors inducing gout attacks. Gouty arthritis is the acute manifestation of gout attacks. A long-term high-uric - acid environment can lead to the deposition of sodium urate crystals in various parts of the body. When the crystals deposit in the kidneys, they form urate stones and cause gouty kidney disease. When they deposit in joints, they form tophi, which will seriously affect the patients’ ability and cause joint swelling, heat, and pain [24], 25]. Therefore, the key to the treatment of gout lies in lowering uric acid, clearing heat, relieving pain, and anti-inflammation. *A. arguta* combines nutrition, health care, and medicinal use all in one. The whole fruit can be used medicinally, and its composition is rich and stable [26]. Meanwhile, it has few side effects, allowing people to consume it safely for a long time. It has multi-components, multi-targets, and can regulate uric acid levels in multiple ways. It can help reduce inflammation, improve hyperuricemia, protect kidney function, and reduce joint inflammation.

Compared with previously reported flavonoid-based agents for gout intervention, the core competitive advantages of AATF extracted from *A. arguta* can be systematically summarized into three dimensions. First, the multi-target synergistic modulation breaks the therapeutic bottleneck of most flavonoids that merely exert a single pharmacological effect (either uric acid-lowering or anti-inflammatory). It enables the concurrent regulation of uric acid production inhibition, uric acid excretion promotion, and inflammatory response suppression, which is more aligned with the pathological essence of gout characterized by the dual pathogenesis of hyperuricemia and inflammatory injury [27]. Second, the component compatibility inherent in natural extracts and prominent safety profile. AATF consists of a spectrum of bioactive constituents including flavonoids, phenolic acids, and polysaccharides, which exert a synergistic effect to enhance the overall therapeutic efficacy; toxicological experiments have verified that it exerts no significant impact on liver and kidney function-related parameters. This unique composition allows for a reduction in the dosage of individual active ingredients, thereby minimizing the potential off-target effects. In contrast, chemically synthesized or individually isolated flavonoid preparations (e.g., high-purity quercetin) are prone to induce gastrointestinal adverse reactions when administered at high doses to achieve a therapeutic effect, while clinically used gout medications such as allopurinol and febuxostat may cause adverse reactions including skin rashes, elevated liver enzymes, and even severe hypersensitivity responses [28], 29]. Third, distinct advantages in resource availability and industrial scalability. *A. arguta* is extensively cultivated in Northeast China, and its fruit processing by-products (e.g., peels and pomace) can be directly utilized as feedstocks for the extraction of AATF. In comparison with imported raw materials such as baicalein and rutin, this locally sourced feedstock confers greater cost controllability and supply chain stability [30]. Collectively, AATF offers a promising novel candidate for the development of natural therapeutics against gout, with advantages that are distinctly superior to those of conventional flavonoid-based drugs and clinical first-line agents [31].

In order to understand the molecular mechanism of AATF treatment gout, we conducted the network pharmacology study, by “drug-ingredients-disease-target-pathway” network, AATF treat gout key active ingredients for skin and gallic acid, these two components have obvious drop uric acid effect, anti-inflammatory, antioxidant, improve the effect of gout arthritis and gout kidney disease [32], 33]. At the same time, it plays a good protective role in kidney damage, which shows that AATF has great development prospects in the treatment of gout.

In predicting the potential targets of AATF for treating gout, we can observe that ALB, TNF, IL-6, TP53, VEGFA, IL-1β, MAPK3, SRC, PTGS2, Signal Transducer and Activator of Transcription 3 (STAT3), CXCL8, Toll – like Receptor 4 (TLR4), PPARG, CCL2, IL-1β, MMP9, Heme Oxygenase 1 (HMOX1), Transforming Growth Factor Beta 1 (TGFB1), Peroxisome Proliferator – Activated Receptor Alpha (PPARA), C-Reactive Protein (CRP), and Xanthine Dehydrogenase (XDH) are centrally distributed among the targets of the active components of AATF. These targets mainly involve biological processes such as immunity, uric acid metabolism, oxidative stress, and inflammation [34]. There is a positive correlation between the aforementioned ingredients and the prognosis and treatment of gout. Among these ingredients, the synthesis of XDH and uric acid excretion are closely related [35]. XDH is a key enzyme in purine metabolism. The prediction results indicate that AATF reduces uric acid, regulates purine metabolism, promotes uric acid excretion, and reduces MSU deposition to achieve the treatment of gouty nephropathy, which may be related to its action on the XDH target [36]. As a danger signal, MSU plays an important role in the onset of gout. When the body is stimulated by MSU, cytokines such as IL-1β, TNF, and IL-6 will increase in the serum and at the injury site, inducing an inflammatory response [37].

**Figure 10:**
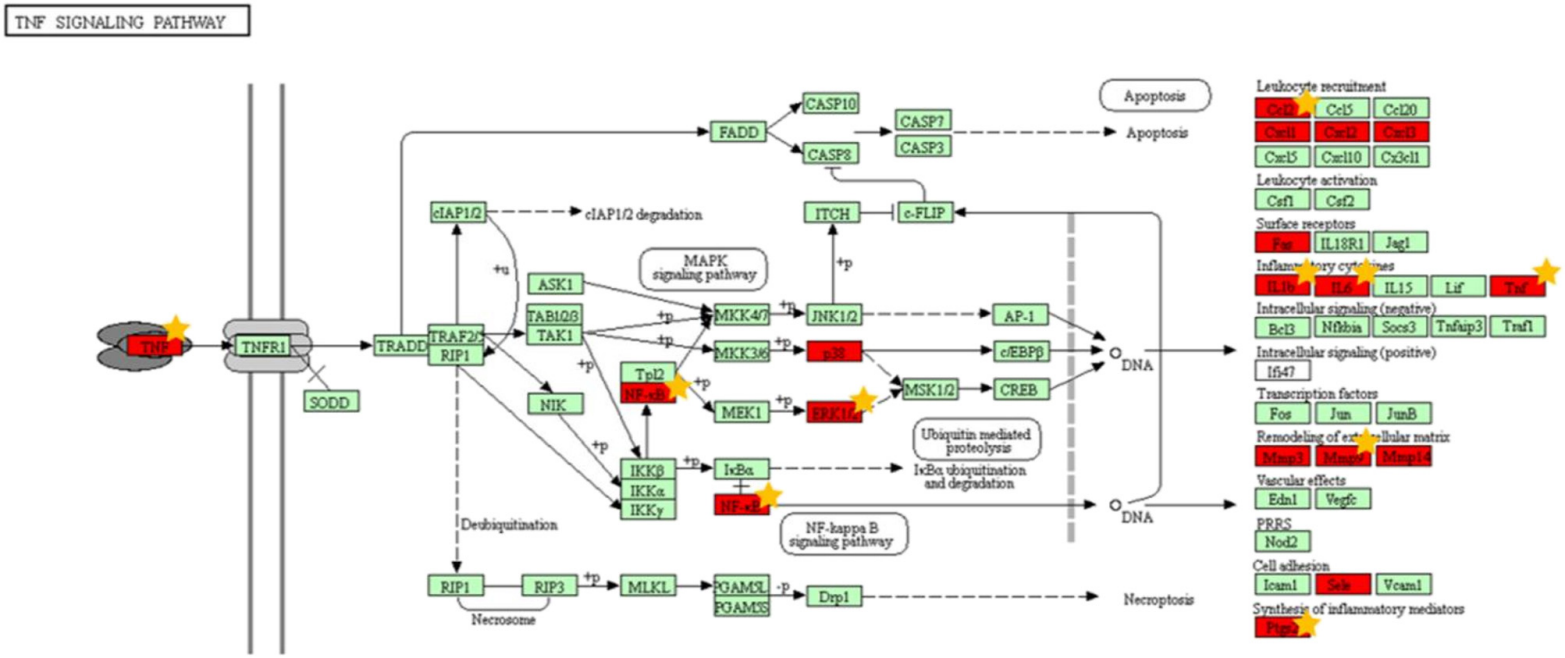
TNF signaling pathway. Red color indicates the targets of AATF for gout treatment, and the yellow pentagram annotation indicates the core targets of AATF for gout treatment.

The KEGG analysis showed that the AATF treatment of gout may be related to the TNF signaling pathway. In the TNF signaling pathway, the Tpl 2-mek 1-ERK kinase cascade, which will lead to the induction of Ptgs two by the Peroxisome Proliferator-Activated Receptor Alpha (CREB/ATF) transcription factor family [38]. COX-2, encoded by Ptgs 2, is involved in the synthesis of prostaglandins, an important mediator of pain, inflammation, and fever [39]. IkB degradation allows a dimeric NF-κB transcription factor, consisting mainly of RelA (p65) and NF-κB 1 (p50) subunits to accumulate in the nucleus, and drives expression of abundant TNF- α, IL-1β and IL-6. MAPK3 encodes the expression of ERK1 protein and MAPK3 is located among our predicted core targets [40], MMP9 is one of the major members of the matrix metalloproteinase family and the literature shows that when ERK1/2 signaling is activated, it increases the expression level of MMP9 and promotes the inflammatory response at the site of injury [41]. The red annotation indicates the active component in AATF for treating gout in the TNF signaling (Figure 10).

To verify the above partial prediction results, we conducted experimental studies on AATF for gout by establishing a rat double model gouty nephropathy combined with gouty arthritis. The results showed that AATF has an obvious uric acid – lowering effect, which can reduce the activity of XOD enzyme, decrease the deposition of uric acid crystals in the kidney, and improve kidney injury. It can also reduce joint swelling in the control rats, relieve the gait state, and exhibit a better anti – inflammatory and analgesic effect. In addition, the protein expression of NF-κB, ERK 1, and MMP 9 in the TNF signaling pathway was significantly reduced, and the content of TNF, IL-1β, IL, IL-6, COX 2 in serum was also significantly reduced. At the same time, the pathomorphological results of the rat ankle joint synovial tissue and kidney tissue showed that AATF could effectively inhibit the proliferation of synovial cells in the ankle joint cavity and prevent the infiltration of inflammatory cells, so as to protect the synovial tissue and improve gout arthritis and kidney injury. The above evidence validates our results in network pharmacology. On the one hand, the feasibility of network pharmacology is verified; on the other hand, the effectiveness of AATF in the treatment of gout has been verified, which is consistent with the efficacy of AATF, suggesting that it may be one of the potential pharmacodynamic substances of AATF against gout. This study provides the basis and support for the clinical use of AATF in treating gout, and it also reflects the characteristics of multi-target and multi-pathways of traditional Chinese medicine in treating diseases.

There is a limitation in our study. Despite the limitations, the integrated findings from network pharmacology prediction and animal experiments still robustly confirm the potential therapeutic efficacy of AATF against gout, with clear mechanistic correlations and translational implications. First, the key bioactive components of AATF identified via network pharmacology were predicted to target the core pathogenic nodes of gout. In silico analysis revealed that these components could XOD activity to reduce uric acid biosynthesis. Simultaneously, AATF was shown to modulate the TNF signaling pathway and downregulate the production of pro-inflammatory cytokines such as TNF-α and IL-1β, thereby exerting a dual therapeutic effect of urate-lowering and anti-inflammation. This multi-target regulatory pattern of AATF aligns well with the complex pathological network of gout, which is characterized by both metabolic disorder and inflammatory cascade reaction.

Second, the animal experimental data provided direct evidence for the efficacy and preliminary safety of AATF. AATF intervention significantly reduced serum uric acid levels, alleviated joint swelling, and ameliorated synovial inflammatory damage in hyperuricemic mice induced by potassium oxonate and hypoxanthine, without causing obvious hepatic or renal dysfunction. Although the acute/short-term animal model used in this study cannot fully recapitulate the clinical features of chronic recurrent gout with tophus formation in humans, the observed pharmacological effects still lay a critical foundation for subsequent in-depth investigations.

Notably, as a natural product with multiple bioactive ingredients, AATF exhibits inherent advantages over single-target chemical drugs in the treatment of gout, especially in managing comorbid metabolic syndrome commonly associated with gout patients. This highlights the unique translational potential of AATF in clinical practice.

In conclusion, the limitations of this study are mainly attributed to the methodological and experimental design constraints at the current stage, rather than the negation of AATF’s therapeutic efficacy. Future research should focus on establishing chronic gout models with MSU crystal deposition, conducting in-depth mechanism studies using gene knockout/knockdown techniques, and evaluating the synergistic effects of AATF combined with clinical first-line drugs. These efforts will further validate and expand the clinical application value of AATF in gout treatment.

## 5 Conclusions

In conclusion, AATF reduced uric acid content and XOD enzyme activity, inhibiting the secretion of inflammatory factors IL-1 β, IL-6, TNF-α, and COX 2, and it reduced the protein expression of NF-κB, ERK 1, MMP 9. This is supposed to be the reason why AATF can relieve kidney injury, joint synovial inflammation, joint pain and swelling in the two-model of gout in rats. The TNF signaling pathway is one of the molecular mechanisms used in AATF treatment treat gout. This study not only elucidates the anti-inflammatory mechanism of AATF, but also provides sufficient and reliable preclinical preliminary evidence for the subsequent development of this natural product into a novel drug for gout treatment.
